# Serotonin transporter availability increases in patients recovering from a depressive episode

**DOI:** 10.1038/s41398-021-01376-w

**Published:** 2021-05-10

**Authors:** Jonas E. Svensson, Cecilia Svanborg, Pontus Plavén-Sigray, Viktor Kaldo, Christer Halldin, Martin Schain, Johan Lundberg

**Affiliations:** 1 Centre for Psychiatry Research, Department of Clinical Neuroscience, Karolinska Institutet, & Stockholm Health Care Services, Region Stockholm, Karolinska University Hospital, SE-171 76 Stockholm, Sweden; 2 Neurobiology Research Unit, Copenhagen University Hospital, Copenhagen, Denmark; 3 Department of Psychology, Faculty of Health and Life Sciences, Linnaeus University, Växjö, Sweden

**Keywords:** Depression, Molecular neuroscience

## Abstract

Molecular imaging studies have shown low cerebral concentration of serotonin transporter in patients suffering from depression, compared to healthy control subjects. Whether or not this difference also is present before disease onset and after remission (i.e. a trait), or only at the time of the depressive episode (i.e. a state) remains to be explored. We examined 17 patients with major depressive disorder with positron emission tomography using [^11^C]MADAM, a radioligand that binds to the serotonin transporter, before and after treatment with internet-based cognitive behavioral therapy. In all, 17 matched healthy control subjects were examined once. Cerebellum was used as reference to calculate the binding potential. Differences before and after treatment, as well as between patients and controls, were assessed in a composite cerebral region and in the median raphe nuclei. All image analyses and confirmatory statistical tests were preregistered. Depression severity decreased following treatment (*p* < 0.001). [^11^C]MADAM binding in patients increased in the composite region after treatment (*p* = 0.01), while no change was observed in the median raphe (*p* = 0.51). No significant difference between patients at baseline and healthy controls were observed in the composite region (*p* = 0.97) or the median raphe (*p* = 0.95). Our main finding was that patients suffering from a depressive episode show an overall increase in cerebral serotonin transporter availability as symptoms are alleviated. Our results suggest that previously reported cross-sectional molecular imaging findings of the serotonin transporter in depression most likely reflect the depressive state, rather than a permanent trait. The finding adds new information on the pathophysiology of major depressive disorder.

## Introduction

Major depressive disorder (MDD) is the leading cause of disability worldwide^1^, but the biological underpinnings of the disorder are still largely unknown. The monoamine hypothesis has been the dominant pathophysiological model for half a century^2^. Aberrations in the monoamine serotonin (5-HT) system is one of the most replicated observations in experimental MDD research^3–5^ and most efficacious pharmacological treatments target the serotonin system^6,7^. However, it is not known if serotonin plays a direct causal role in the pathophysiology of depression, as suggested by the classical interpretation of the monoamine hypothesis;^5^ or if change in 5-HT activity is part of a salutogenic response to stress, enhancing adaptive responses to adverse conditions^8,9^. Nor is it known if aberrations in the serotonin system are trait-like, or if they are temporary, only present during the time of the illness^10,11^. To answer these basic questions is of key importance for the development of better MDD treatment options.

The serotonin transporter (5-HTT) has received a lot of attention in MDD research, in part since it is the primary mechanism to decrease extracellular 5-HT^12^, but also because it is the target of the most commonly used antidepressant drugs^13^. Positron emission tomography (PET) have been used to study 5-HTT in depression. Results from individual studies differ, showing lower binding in cortical and subcortical regions^14–17^, but also higher binding^18^. A meta-analysis has shown reduced 5-HTT binding in the midbrain and amygdala^19^, indicating a general trend towards lower cerebral 5-HTT availability in patients suffering from MDD^20^.

The dorsal and median raphe nuclei, located in the brainstem, are the main sites for cell bodies of serotonergic neurons^12^ and has the highest concentration of 5-HTT in the brain^21^. While both nuclei project axons to a wide array of partially overlapping cerebral targets the pattern of projections differ^22^. The median raphe has been suggested to be of particular interest with regards to MDD^23,24^. The raphe nuclei are small structures that are not observable on a magnetic resonance image (MRI). This makes it challenging to examine with PET, especially using imaging systems with low spatial resolution. Results from meta-analyses of molecular imaging data suggest low 5-HTT availability also in raphe in MDD, but the interpretation is hampered by methodological drawbacks in the many different methods used for spatial definition.

In contrast to the relatively large number of cross-sectional (i.e., patient-control) PET-studies on MDD and 5-HTT there is a lack of studies examining the longitudinal course of 5-HTT availability in depression. It is thus unclear if the putative lower level of 5-HTT represent a “trait”, i.e., a risk factor for MDD chronically present, or a “state”, i.e., present only around the time of the depressive episode. Since most pharmacological treatment options against MDD affects the serotonergic system, it is difficult to interpret the results from pre-post molecular imaging studies using drugs to understand the natural progression of the disorder. Cognitive behavioral therapy (CBT), on the other hand, has been shown to be as effective as pharmacological treatment of depression^25^. Internet delivered CBT (ICBT) has been found to be as effective as face-to-face CBT, and is available as an option in regular health care in many countries^26,27^. ICBT has standardized treatment modules, ensuring that all subjects get as close to the same intervention as possible and is thus a suitable intervention in a study of the longitudinal course of 5-HTT availability in MDD.

The primary aim of this study was to address the trait-state question of MDD biology by examining if the 5-HTT availability changes as subjects improve from a depressed state. The secondary aims were to compare the 5-HTT availability in the brain of depressed individuals with healthy controls; and to apply a novel approach to delineate the raphe nuclei and examine these regions separately using a high-resolution PET system.

## Materials and methods

### Subjects

The study was approved by the Research Ethics Committee in Stockholm, Sweden, and the Radiation Safety Committee at Karolinska University Hospital, Stockholm. All subjects gave verbal and written informed consent before participation.

In all, 17 patients with MDD and 17 healthy controls were included in the study. This sample size was decided on based on test–retest data for the applied radioligand^28^. All subjects were recruited by advertisement in local newspapers. Patients were assessed at the Internet Psychiatry clinic, which is part of the Region Stockholm University Health Care^27^. Diagnoses were given based on a full psychiatric assessment by a psychiatrist or a resident physician supervised by a psychiatrist, using the Mini-International Neuropsychiatric Interview (M.I.N.I.)^29^. Post-treatment, the supervising psychiatrist assessed patients at a physical encounter. All subjects were healthy according to clinical interview, MRI of the brain, physical examination, and standard laboratory tests. Patients had an ongoing major depressive episode diagnosed using DSM-IV criteria^30^, with at least one prior episode of MDD. Montgomery Asberg Depression Rating Scale^31^ (MADRS) was used to assess depression severity. Patients with a score above 18 and below 35 were eligible for inclusion. No ongoing psychopharmacological treatment of MDD was allowed. The subject with most recent exposure to antidepressant drug treatment had this discontinued 8 months prior to study inclusion.

The control subjects did not fulfill criteria for MDD, or any other diagnosis according to M.I.N.I., and had no history of psychiatric illness. Controls were matched to the patients for sex, age (±3 years) and intellectual ability (±1 standard deviation (SD) in the Matrix Reasoning subtest of WAIS-IV); (Table 1).

**Table 1:** Demography and radioactivity data.

	Patients	Controls	*P* value^a^
Gender, *n*			
Female	13	13	–
Male	4	4	–
Age, years (mean ± SD)	47 ± 13	47 ± 14	–
WAIS, matrix reasoning^b^ (mean ± SD)	12.4 ± 2.9	12.7 ± 3.2	–
Current MDD episode duration, months	15 ± 13	–	–
Family history of MDD, *n* (%)	7 (41%)	–	–
Prior MDD episodes, *n* (%)			
1–2	8 (47%)	–	–
>2	9 (53%)	–	–
Prior CBT, *n* (%)	9 (53%)	–	–
Previous AD, *n* (%)	9 (53%)	–	–
Time since AD, months (min–max)	8–204	–	–
Comorbid psychiatric diagnosis^c^, *n* (%)	6 (35%)	–	–
Injected radioactivity, MBq (mean ± SD)			
PET1	424 ± 82	408 ± 85	0.57
PET2	406 ± 91	–	0.56
Molar radioactivity, GBq/μmol (mean ± SD)			
PET1	216 ± 65	231 ± 134	0.67
PET2	200 ± 81	–	0.52
Injected mass, μg (mean ± SD)			
PET1	0.58 ± 0.19	0.93 ± 1.28	0.28
PET2	0.68 ± 0.38	–	0.33

*AD* antidepressant, *CBT* Cognitive Behavioral Therapy, *MDD* major depressive disorder.

^a^Calculated using independent *t* test.

^b^Raw score standardized for age.

^c^Generalized anxiety disorder; social anxiety disorder; panic disorder; hypochondriasis.

### Study design

**Fig. 1:**
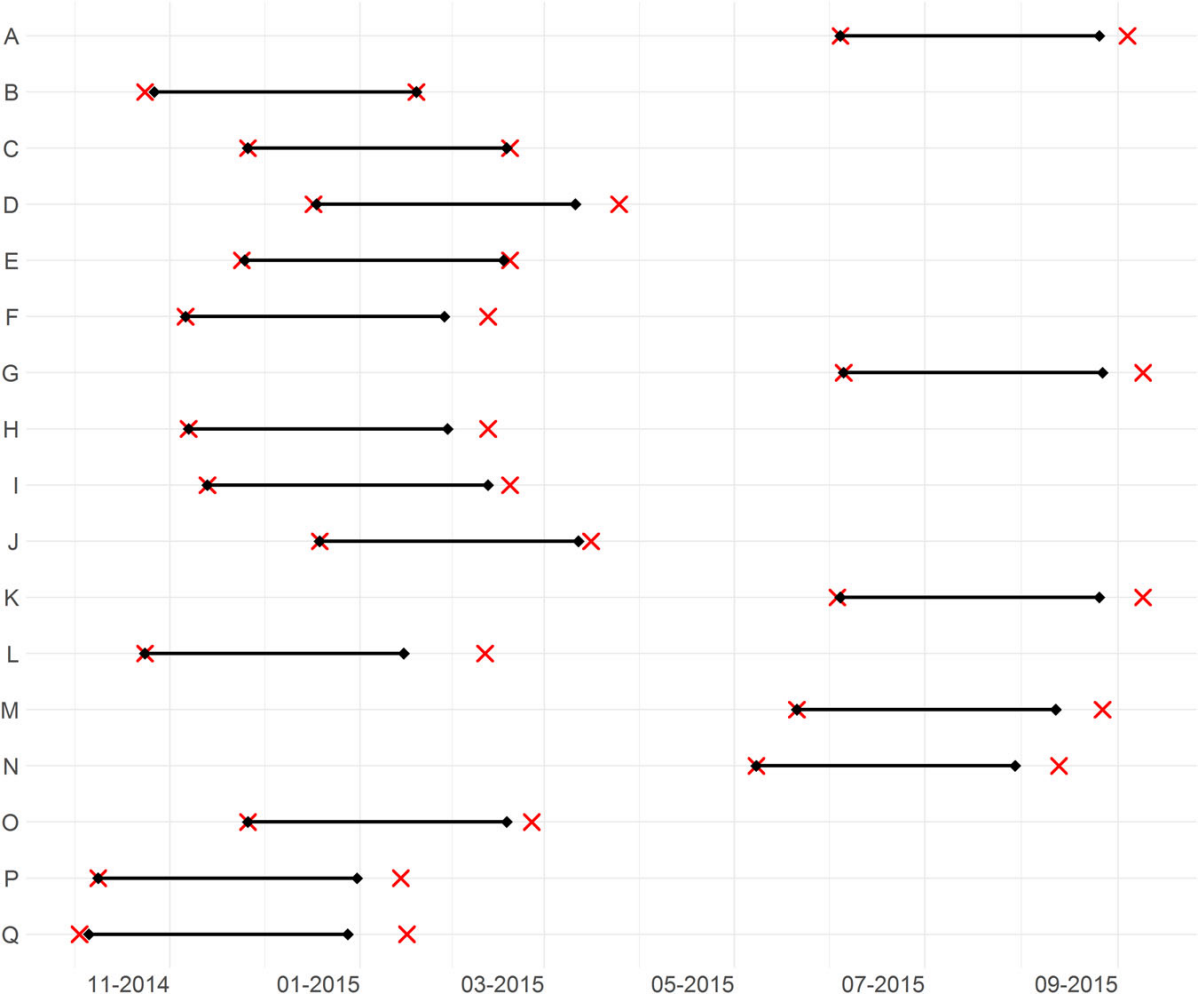
Timelines for study participation of all MDD patients. Dates for initiation and completion of cognitive behavioral therapy (black diamonds connected with horizontal line), and PET1 and PET2 (red crosses). One row per patient, letters corresponding to Fig. 3A.

After the first PET examination (PET1) ICBT was initiated followed by a second PET examination (PET2) after treatment completion. Self rated MADRS^32^ (MADRS-S) was measured weekly. Clinical global rating scale Improvement (CGI-I) was assessed at follow up (details on rating scales in Supplement 1). The average treatment duration was 11 weeks and time between PET1 and PET2 was 12–16 weeks (Fig. 1). Controls were recruited separately; they did not receive any treatment and were examined once with PET.

### Cognitive behavioral therapy

The ICBT treatment protocol applied in this study has been tested in randomized studies^33^, and shown to be effective^34^. The main component of the treatment program is 10 text modules, covering specific themes such as psychoeducation, behavioral activation, cognitive restructuring, and relapse prevention. Each module ends with a homework assignment. Throughout the treatment, all participants are assigned a psychologist who supervises the progress and provides individual feedback.

### MRI and PET experimental procedure

T1-weighted MRI images were acquired using a 3T GE Signa system (GE Medical Systems, Chicago, Illinois, USA). All subjects were examined using a high-resolution research tomograph (Siemens Molecular Imaging, USA) with a maximum spatial resolution of ~2 mm full-width-half-maximum^35^. Transmission scans were performed prior to each PET measurement to correct for signal attenuation.

[^11^C]MADAM, a radioligand suitable for quantification of the serotonin transporter, was synthesized as described previously^36^. In each PET-experiment a saline solution containing [^11^C]MADAM was injected into a antecubital vein as a bolus (<10 s). The cannula was then flushed with 10 mL saline. Injected radioactivity, molar radioactivity and injected mass are reported in Table 1. Emission data were acquired continuously for 93 min, and subsequently binned into 38 consecutive time frames using the following frame definitions: nine 10 s, two 15 s, three 20 s, four 30 s, four 1 min, four 3 min, and twelve 6 min frames.

### Image preprocessing and quantification

Dynamic PET images were corrected for head motion using a between-frame-correction algorithm implemented in SPM12 (Wellcome Department of Cognitive Neurology, University College, London, UK) where frames were realigned to the first six-minute frame. Using SPM12 the T1-weighted MR-images were then co-registered to a time-weighted summated PET-image. To derive regional time-activity curves (TACs), the resulting co-registration matrix was used to project regions of interest (ROIs) on the realigned dynamic PET-image.

From the time-activity curves, binding potential with respect to non-displaceable uptake (*BP*_ND_) was calculated for each ROI using the non-invasive Logan plot^37^ fitted with multilinear regression^38^, with t* = 45 min, corresponding to eight frames. The model requires the reference region efflux rate constant (k2′) as an input. This was set to the k2′ value from putamen^39^, derived using the simplified reference tissue model^40^, resulting in values between 0.04 and 0.11, in line with values derived using arterial input function^37^. Cerebellar gray matter was defined as described previously^41^ and used as reference region^42^.

Parametric images were generated using the 3D stationary wavelet aided parametric imaging (WAPI) procedure, where the non-invasive Logan plot, fitted with multilinear regression, is applied on TACs from individual voxels^43,44^. For visualizations, the parametric images were registered to MNI-space^45^ and averaged across individuals.

### Regions of interest

FreeSurfer (version 6.0, http://surfer.nmr.mgh.harvard.edu/)^46^ was used to delineate brain regions on the T1-weighted MRIs. We tested two regions of interest: (i) median raphe nuclei, and (ii) a composite region consisting of anatomical structures where the [^11^C]MADAM signal-to-noise ratio was deemed acceptable: amygdala, anterior cingulate gyrus (ACC), posterior cingulate gyrus (PCC), caudate, hippocampus, insular cortex, putamen, and thalamus. The composite region was created using weighted standardization, with weights derived from the volume and variance in *BP*_ND_ for each of the included ROIs (see Supplement 1 for details).

The reasons for creating a composite ROI were: (i) 5-HT proteins examined with PET have shown high interregional correlations^47^, supporting the idea of a central regulation of the expression of serotonergic proteins from the raphe nuclei. By extension, a putative change in 5-HTT could be expected globally in the brain. (ii) Previous patient-control molecular imaging studies of 5-HTT in MDD have implicated several different brain regions^14–17,48^. (iii) Combining many small regions into one large increase the statistical power through reduction both of noise and the number of comparisons.

**Fig. 2:**
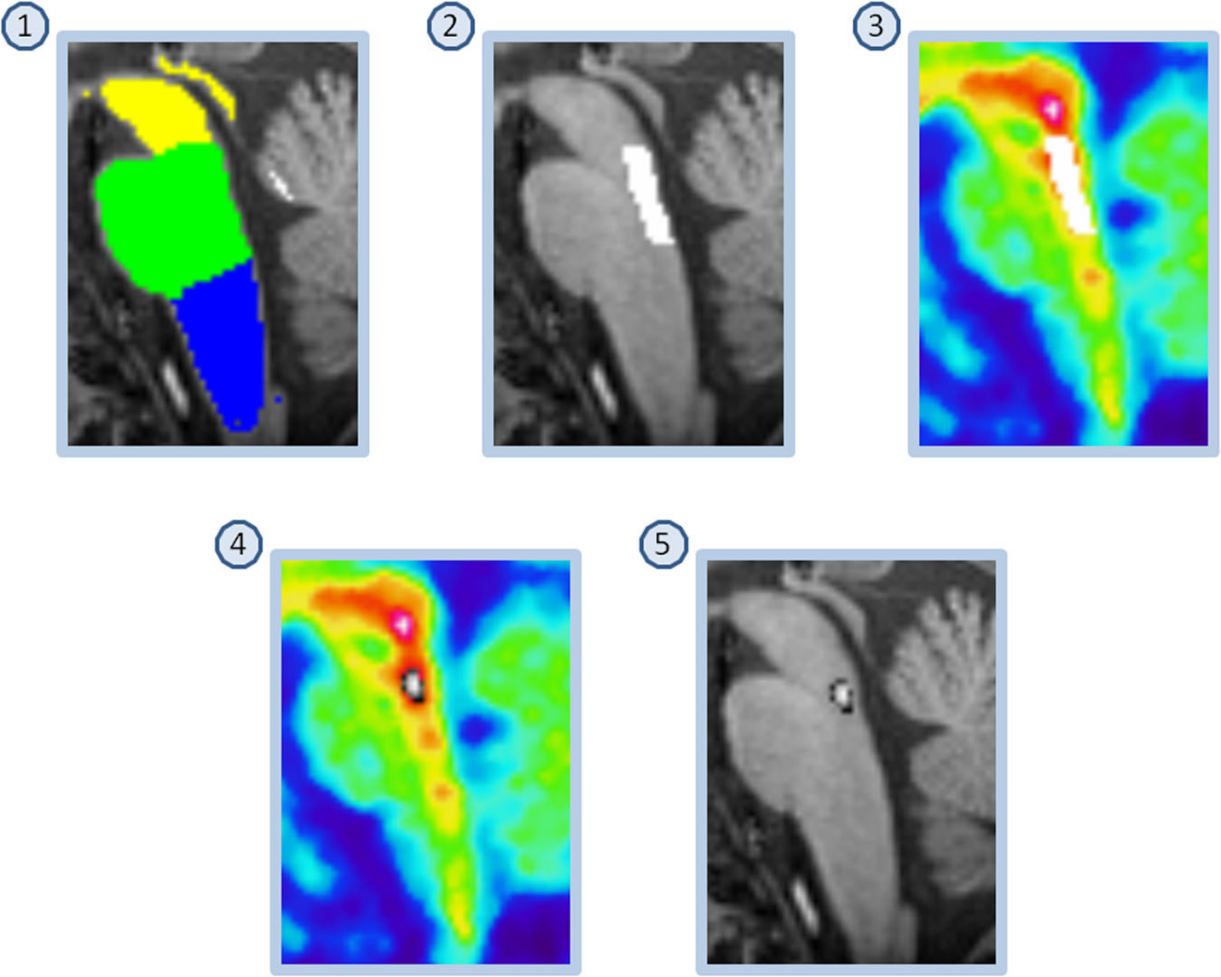
Delineation of region preferentially sampling median raphe. (1) FreeSurfer Brainstem Substructures. (2) The pons mask (green) was trimmed, keeping the most dorsal 5 voxels. (3) The resulting mask was overlaid on the time-weighted summated PET-image. (4) Median raphe is the structure with highest [^11^C]MADAM binding within the boundaries of the trimmed pons mask; the most intense PET-voxel was located and in an iterative process the voxel with highest intensity adjacent to the initial voxel was added until 65 voxels were collected. (5) The resulting mask overlaid on the MR-image. The same procedure was used to delineate a region preferentially sampling dorsal raphe except for using the midbrain mask (yellow in panel 1) and 116 voxels.

ROIs preferentially sampling the dorsal- and median raphe respectively were created using a version of a previously described semi-automatic method^44,49^, here fully automated (Fig. 2, see Supplement 1 for details). For each individual, the resulting masks were applied to the WAPI-images to calculate a *BP*_ND_ value.

### Preregistration

Following recent recommendation for PET studies^50^, the image analysis and statistical plan was preregistered at AsPredicted.com (Supplement 2). The preregistration was submitted after collection of data was completed, but before any analysis of PET-data was performed. Deviations from the preregistration were as follows: (i) Slow radioligand kinetics were observed in pallidum in several controls and patients. A central assumption of the model used for quantification of [^11^C]MADAM binding is reversible kinetics of the radioligand. Since no clear peak of the time activity curve was observed in eight subjects, pallidum was removed from the composite region in the main analysis. (ii) In one patient the *BP*_ND_ values calculated in the second PET examination were unrealistically high (~4 standard deviations higher than the average). This subject was excluded from the longitudinal analysis, but the baseline examination was included in the cross-sectional analysis. In Supplement 1, we report results including this outlier and with pallidum included in the composite region, i.e., verbatim the preregistration (Supplement 1, Fig. S1 and S2; Table S1). All analyses that were part of the preregistration plan are presented as confirmatory. Analyses that were not part of the preregistration plan are presented as exploratory.

### Statistics

#### Confirmatory analyses

Change in MADRS-S was assessed using a multilevel model for repeated measures. Subjects were fitted over time with varying intercepts and slopes. CGI-I data was tested against “4” (i.e., “no change”) using the Wilcoxon one-sample signed rank test.

Paired t-test was used to assess differences in *BP*_ND_ within patients before and after CBT, as well as between patients at baseline and their matched control subjects. For the cross-sectional analysis of the composite ROI, the preregistered prediction was that healthy control subjects should show higher *BP*_ND_ compared to patients, hence this test was one sided. All other tests were two sided. Alpha was set to 0.05 for all statistical tests.

In order to estimate the magnitude of difference between patients before and after treatment, and between patients and controls, the ratio of paired measurements was calculated for *BP*_ND_ of all ROIs that were part of the composite region. The median ratio was then calculated for each patient or patient-control pair. The average percentage difference between groups was reported.

#### Multiple comparisons

The longitudinal and cross-sectional analyses were considered separately. For both analyses two tests were performed: median raphe and the composite region. As per the preregistration, we examined the between-individual correlation between the difference score of the composite region and median raphe to decide if alpha correction was warranted. The correlation in the cross-sectional data was low (*r* = 0.25), indicating low dependence. For this reason, Bonferroni correction was applied. The correlation in the longitudinal analysis was high (*r* = 0.77), and no correction of alpha was made for this analysis.

#### Exploratory analysis

Paired two-tailed *t*-test was used for analysis of *BP*_ND_ values extracted from individual brain regions, both in the longitudinal and cross-sectional data.

To examine if change in MADRS-S was associated to change in [^11^C]MADAM *BP*_ND_ between PET1 and PET2, we extracted the maximum likelihood estimates of the subject specific slopes from the MADRS-S multilevel model. These were then entered as an independent variable into a regression model, predicting *BP*_ND_ from PET2 while controlling for *BP*_ND_ from PET1. Pearson’s correlation coefficient was calculated to examine if baseline *BP*_ND_ was associated with baseline MADRS-S score.

Effect sizes were quantified using Cohen’s dz, a paired version of the classical Cohen’s d where the standard deviation of the difference score is used as denominator^51^.

## Results

### Confirmatory analysis

#### Longitudinal data

**Fig. 3:**
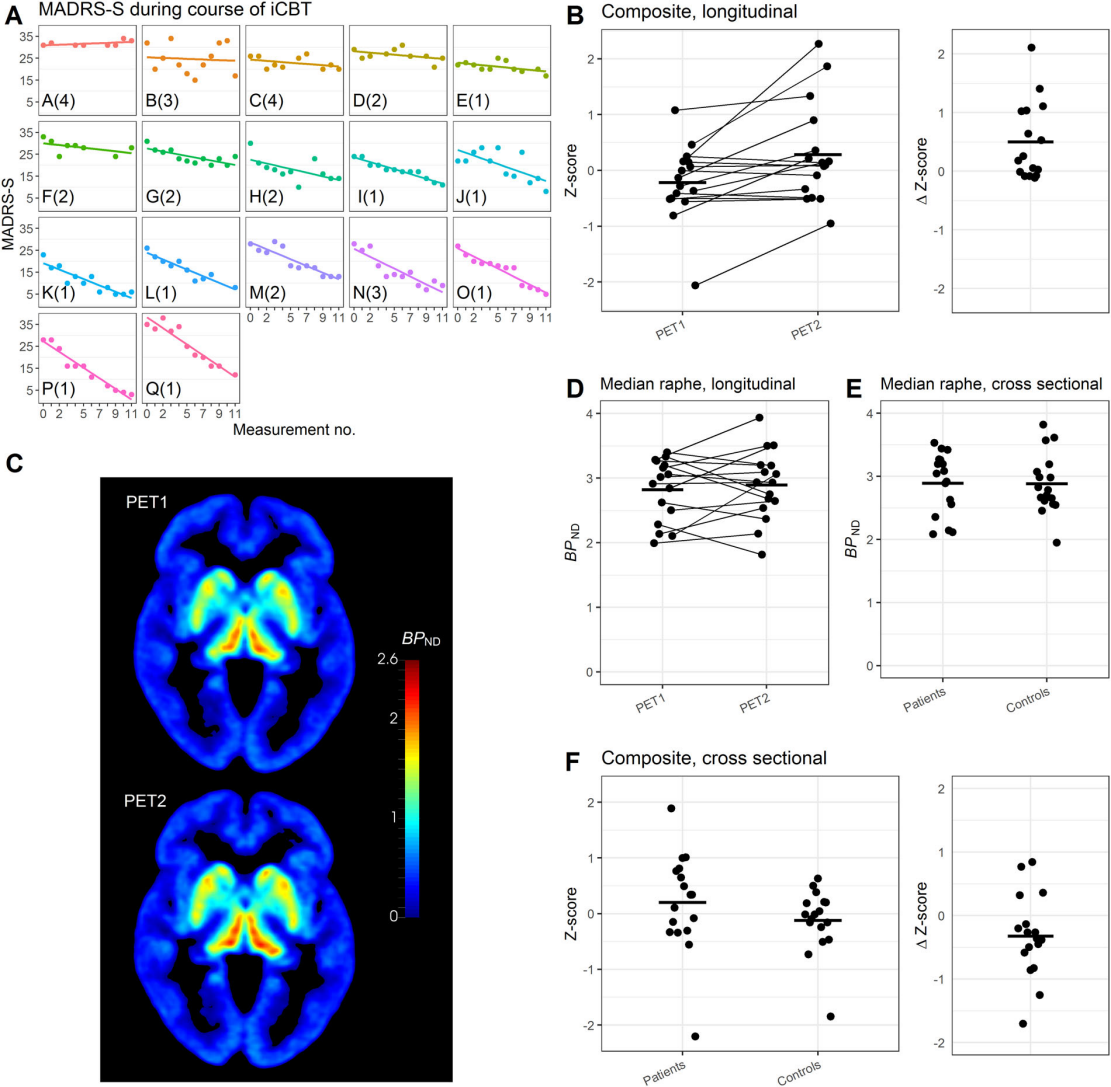
MADRS-S and PET data. In **A** MADRS-S measurements during the course of ICBT. Each cell represents one patient, (*n* = 17), letters in lower left corner corresponds to the letters in Fig. 1, numbers in parenthesis is CGI-I. In **B** longitudinal [^11^C]MADAM data (*n* = 16) for the composite region, PET1 (baseline) and PET2 (after treatment). Right panel shows difference scores between scans. In **C** mean parametric image of patients at baseline (upper image) and after treatment (lower image; *n* = 16), see Supplement 3 for a 3D movie of the same data. In **D** longitudinal data, median raphe, *BP*_ND_ at PET1 (baseline, left) and PET2 (after treatment, right). In **E** cross-sectional data, median raphe, *BP*_ND_ for patients with MDD at baseline (left) and healthy controls (right). In **F** cross-sectional [^11^C]MADAM data (*n* = 17 + 17) for the composite region, comparing patients with MDD at baseline to matched healthy controls. Right panel shows difference scores between matched pairs.

MADRS-S decreased, from 27.9 ± 3.8 (mean ± SD) at start of the ICBT treatment, to 14.9 ± 8.6 at completion (*p* < 0.001; Fig. 3A). The CGI-I data showed that 13 of 17 patients were classified as either “much” or “very much” improved. The test against “no change” was significant (*p* < 0.001), supporting an improvement in symptoms.

*BP*_ND_ increased on average 10% in the composite region following ICBT; PET 1 Mean *Z*-score = −0.22 ± 0.68, PET2 Mean *Z*-score = 0.28 ± 0.89, *t*(15) = −3.01, *p* = 0.01, 95% CI [−0.85, −0.15], Dz = 0.75. Binding in median raphe showed no change following treatment; PET 1 Mean *BP*_ND_ = 2.81 ± 0.48, PET2 Mean *BP*_ND_ = 2.89 ± 0.53, *t*(15) = −0.67, *p* = 0.51, 95% CI [−0.31, 0.16], Dz = 0.17 (Fig. 3B–D, Supplement 3 for a 3D movie).

#### Cross-sectional data

There was no evident difference in *BP*_ND_ between patients at baseline and healthy controls. [^11^C]MADAM binding in the composite region was on average 4% lower in controls compared to patients. The average Z-score for the composite region was −0.12 ± 0.57 for controls and 0.2 ± 0.89 for patients, providing no evidence for a difference between the groups, *t*(16) = −2.03, *p* = 0.97 (one sided), 95% CI [−0.60, Inf], Dz = 0.4 (Fig. 3C). For the median raphe, the average *BP*_ND_ was 2.88 ± 0.47 for controls and 2.89 ± 0.49 for patients, *t*(16) = −0.06, *p* = 0.95, 95% CI [−0.31, 0.29], Dz = 0.01 (Fig. 3E, F).

### Exploratory analysis

In the longitudinal data frontal cortex, parietal cortex, ACC, PCC, insula, hippocampus and thalamus show higher *BP*_ND_ after ICBT. In the cross-sectional data hippocampus show higher *BP*_ND_ in patients at baseline compared to controls (Supplement 1, Table S2 and S3).

Change in MADRS-S was not a significant predictor of PET2 *BP*_ND_, when controlling for PET1 *BP*_ND_ (B1 = 0.007, SE = 0.27, *t* = 0.03, *p* = 0.98). We also included an interaction effect between baseline *BP*_ND_ and MADRS-change. However, neither MADRS-change (*p* = 0.82), nor the interaction term (*p* = 0.65) was significant. No significant association was observed between baseline MADRS-S and PET1 BPND, *r*(15) = −0.24, *p* = 0.36.

## Discussion

Here we show an increase of cerebral serotonin transporter availability in patients with MDD after symptom improvement following engagement in internet delivered CBT. This is, to our knowledge, the first published PET study assessing 5-HTT in patients with MDD before and after non-pharmacological treatment, a design informing on the within-subject change in 5-HTT availability during and after a depressive episode. The observed increase of available 5-HTT indicates a degree of plasticity in the serotonin system in vivo in MDD patients. This suggests that previously reported 5-HTT PET findings in depression are likely to reflect a temporary state rather than a trait.

The observed increase in [^11^C]MADAM binding can have several causes, such as (i) lower levels of serotonin in the synapse, freeing up more 5-HTT for [^11^C]MADAM to bind to; (ii) an increase of the number of synapses expressing 5-HTT; or, (iii), an increase of the 5-HTT concentration in existing synapses. Explanation (i) is unlikely since 5-HTT radioligands is insensitive to change in endogenous serotonin levels in humans^52^. As for (ii), there is evidence of synaptogenesis as part of recovery of depression^53–55^. However, by extension, MDD patients could then be expected to have lower concentration of all 5-HT proteins due to fewer 5-HT synapses compared to healthy controls. Though this might be the case for some proteins (e.g., 5HT1B^56^), it does not seem to be a general finding^57^. We hence suggest, that out of the three explanations above, an upregulation of 5-HTT expression, is the most likely reason for the observed increased [^11^C]MADAM binding.

It has been suggested that the principal function of brain serotonin is to enhance adaptive responses to adverse conditions through improving an individual’s stress tolerability^8,9^. In a longitudinal PET study of individuals resilient to seasonal depression, 5-HTT binding was shown to decrease in the winter^58^. This was interpreted as 5-HTT downregulation mediating resilience to the environmental stress of winter. This model find some support in animal data, where a PET-study in mice using corticosterone treatment to induce a state of chronic stress shows a general decrease in 5-HTT availability^59^. According to this model, environmental stress would decrease cerebral 5-HTT availability as (part of) an adaptive response. Some individuals will still develop MDD, after which, hypothetically, either continual environmental stress or the stress inherent to the depressive state will keep 5-HTT levels low. Pharmacological treatment inhibiting 5-HTT could here be viewed as enhancing the innate 5-HTT reduction. As the individual recovers, either due to spontaneous remission or treatment, e.g., CBT, 5-HTT levels could be expected to increase towards premorbid levels. Though our study design does not allow confirmation of a causal explanation, the results, showing an increase in 5-HTT availability after treatment, are in line with this model.

Change in [^11^C]MADAM binding in individual brain regions follows the same pattern as the composite ROI (Supplement 1, Table S2), with binding increasing after treatment. Most cortical regions were not included in the composite region due to an a priori decision aiming to maximize signal-to-noise. In the exploratory analysis we observed an increase in [^11^C]MADAM *BP*_ND_ of a magnitude similar to the composite region in frontal and parietal cortex but not temporal cortex.

Meta-analyses of cross-sectional 5-HTT PET-studies, published after the design of the present work, have shown higher binding in some brain regions in healthy controls compared to MDD patients. Standardized effect sizes ~0.5 has been reported^19,20^. We instead observed a (non-significant) numerical difference in the opposite direction both in the composite region and in individual brain regions (Supplement 1, Table S3). However, it should be noted that to reliably detect effects of *D* = 0.5, large sample sizes of ~100 subjects per group is needed.

We did not observe a significant correlation between the degree of improvement in MADRS-S and the amplitude of change in [^11^C]MADAM binding. Given our sample size, not much can be inferred from a lack of a significant association; we had adequate power only to detect a true correlation of larger magnitude (*r* > 0.65). To our knowledge, no consistent pattern between symptom ratings in MDD and 5-HTT availability have been reported^20^. It is perhaps not to be expected that MADRS-S total score, which is a composite of many different symptoms in depression, should be strongly explained by the availability of one single protein.

In the analysis of median raphe, no difference was observed between patients and controls, nor was any change in *BP*_ND_ detected in the longitudinal analysis. The analysis of dorsal raphe showed similar results (Supplement 1, Table S2 and S3). Due to the small size of dorsal and median raphe the TACs are noisy, making it hard to assess the kinetics for individual examinations. When deriving standardized uptake curves, averaged across subjects, it can be observed that time for maximum activity (*T*_max_) not is reached within the time frame of data acquisition, implying that the binding is not reversible during the time span of the PET experiment (Supplement 1: Figs. S3 and S4). If transient equilibrium is not reached true differences between populations could be difficult to detect. This should be taken into consideration when interpreting the results.

A limitation in this study is the fact that PET examinations of patients were performed between October 2014 and September 2015, while healthy controls were examined between May 2016 and October 2017. To the best of our knowledge all conditions around the PET-examinations were unchanged during this time period.

Only the patients were examined twice with PET and [^11^C]MADAM. This may be viewed as a limitation of the study. But as the research question was whether 5-HTT density in the brain changes as a depressed individual becomes less depressed, the cause of the improvement was not of interest. Thus, a randomized placebo-controlled design was deemed inappropriate. Indeed, it is likely that, in addition to the effects of the ICBT treatment, there was an improvement caused by time, but this is desirable and adjusting for it in the study design would do naught besides reduce statistical power. Another alternative would be to examine the controls twice – in effect a test-retest design on healthy individuals. However, there is a priori no reason to believe that the average concentration of 5-HTT in a group of healthy individuals should systematically change in any direction over a period of 11 weeks. It is also unlikely that any such difference would be big enough as to have any detectable effect in a study of *N* = 17. Additionally, at least seven test-retest studies have been published on 5-HTT binding radioligands^39,60–64^, including a study on [^11^C]MADAM with 4–8 weeks between test and retest published by our group^28^. Unsurprisingly, though point estimates of the difference scores differ between brain regions and studies, no systematical direction can be discerned. Since PET-examinations are both costly and entails exposing individuals to radiation we decided that the additional information provided by a second PET measurement of the controls not to be worth the cost

Here we demonstrate that patients in an MDD episode increase serotonin transporter availability after alleviation of depressive symptoms. Our results suggest plasticity in the serotonin system with regards to depression.

## Supplementary information

Supplement 1

Supplement 2

Supplement 3
