# Supplementary material for: Serotonin transporter availability increases in patients recovering from a depressive episode
Source: Transl Psychiatry. 2021 May 10;11:264. doi: 10.1038/s41398-021-01376-w (PMC8110529; doi:10.1038/s41398-021-01376-w)
Supplement: Supplementary file 1 — Supplement 1 [file 41398_2021_1376_MOESM1_ESM.docx]

**Serotonin transporter availability increases in patients recovering from a depressive episode**

Supplementary information

Rating scales

MADRS-S measures nine clinical characteristics of depression and the total score ranges from 0 to 54 (Montgomery and Åsberg, 1979; Svanborg and Åsberg, 1994). Scores of 13-19 are considered as mild depression, 20-34 as moderate, and 35 and above as severe depression. The MADRS-S is highly correlated (r=0.83) to the Beck Depression Inventory (Beck et al., 1961). MADRS-S data was gathered at initiation of treatment, once per week during the treatment period, and at the conclusion of treatment, totaling 12 measurements points.

Clinical global rating scale Improvement (CGI-I) is a clinician rated seven-point scale of the patient’s change from the initiation of treatment. 1=very much improved since the initiation of treatment; 2=much improved; 3=minimally improved; 4=no change; 5=minimally worse; 6= much worse; 7=very much (Busner and Targum, 2007).

Composite region weighting scheme

For each brain region of interest (ROI), a vector containing the *BP*_ND_ values from all examinations (e.g., for longitudinal analysis 34 values, two from each patient) was created. This vector was then standardized by setting the mean to zero and the SD to 1, hence yielding a z-score. Each examination (e.g., subject 1, PET1) then had eight standardized measurements, one for each ROI. A weighted average z-score was calculated for each examination, with weights derived from the volume and variance in *BP*_ND_ for each ROI. The weights were created as follows: For each ROI the following parameters were extracted from the parametric image volume: number of voxels (N); mean *BP*_ND_; standard deviation (SD) of the *BP*_ND_ estimate. The standard error of the mean (SE) was calculated according to:$SE=\frac{SD}{\surd N}$. The weight for each ROI was then calculated as: $\frac{\bar{{BP}_{ND}}}{SE}$. Each weight was divided by the sum of all the weights in that examination to create a vector with the sum of 1. This resulted in one average z-score per examination. The same method was used both for the cross-sectional and longitudinal analysis.

Delineation of raphe nuclei

The FreeSurfer tool Brainstem Substructures (Iglesias et al., 2015) was used to extract a pons ROI, wherein median raphe is situated, and a midbrain ROI wherein dorsal raphe is situated. These ROIs were automatically trimmed, only keeping the most dorsal 5mm section. The resulting masks were applied to a time-weighted summated PET-image, smoothed using a 3-voxel median filter. Within the masks the PET-voxel with the highest count was identified and in an iterative process the voxel with highest count adjacent to the initial voxel was added. This process continued until the DR mask constituted 116 voxels and the median raphe mask 65 voxels (Beliveau et al., 2015). For the longitudinal analysis the masks from PET1 and PET2 were combined to create one mask applied to both examinations. For the cross-sectional analysis the mask from PET1 of both patients and controls was used.

**Supplementary Figure 1**. [11C]MADAM data, before and after cognitive behavioral therapy against major depressive disorder. In B) the difference score (PET2-PET1). In this analysis pallidum was included in the composite region and the outlier that was excluded in the main analysis is present.

**Supplementary Figure 2**. [11C]MADAM data, patients with major depressive disorder and healthy controls In B) the difference score (matched controls - patients). In this analysis pallidum was included in the composite region.

| **Supplementary Table 1.** [^11^C]MADAM data, longitudinal and cross sectional analysis (pallidum included) | | | | | | | | | | | | | |
| --- | --- | --- | --- | --- | --- | --- | --- | --- | --- | --- | --- | --- | --- |
| *Region* | *PET1 (Z-score)* |  | *PET2 (Z-score)* |  | *Difference* |  | *Paired t-test* | | | | | | |
|  |  |  |  |  |  |  |  |  |  |  |  |  |  |
|  | *Mean ± SD* |  | *Mean ± SD* |  | *Mean ± SD* |  | DF |  | t statistic |  | p value |  | CI (95%) |
|  |  |  |  |  |  |  |  |  |  |  |  |  |  |
| Copmposite CS | 0.2± 0.88 |  | -0.13± 0.57 |  | -0.33± 0.68 |  | 16 |  | -2 |  | 0,063 |  | [-0.68± 0.02] |
| Copmposite long | -0.25± 0.54 |  | 0.3± 1.08 |  | 0.54± 0.88 |  | 16 |  | -2.55 |  | 0,021 |  | [-0.99± -0.09] |
|  |  |  |  |  |  |  |  |  |  |  |  |  |  |
| CS, Cross sectional (PET1 is patients and PET2 is controls); long, longitudinal (PET1 is baseline and PET2 after cognitive behavioral therapy) | | | | | | | | | | | | | |

| **Supplementary Table 2.** [^11^C]MADAM data pre- and post cognitive behavioral therapy against MDD | | | | | | | | | | | | | |
| --- | --- | --- | --- | --- | --- | --- | --- | --- | --- | --- | --- | --- | --- |
| *Region* | *Scan 1 (BP_ND_)* |  | *Scan 2 (BP_ND_)* |  | *Difference* |  | *Paired t-test* | | | | | | |
|  |  |  |  |  |  |  |  |  |  |  |  |  |  |
|  | *Mean ± SD* |  | *Mean ± SD* |  | *Mean ± SD* |  | DF |  | t statistic |  | p value |  | CI (95%) |
|  |  |  |  |  |  |  |  |  |  |  |  |  |  |
| Frontal cortex | 0.22± 0.07 |  | 0.27± 0.09 |  | 0.04± 0.06 |  | 15 |  | 3.16 |  | 0.006 |  | [0.01± 0.07] |
| Occipital cortex | 0.28± 0.08 |  | 0.3± 0.08 |  | 0.02± 0.05 |  | 15 |  | 1.76 |  | 0.099 |  | [-0.01± 0.05] |
| Parietal cortex | 0.24± 0.07 |  | 0.3± 0.1 |  | 0.06± 0.06 |  | 15 |  | 3.76 |  | 0.002 |  | [0.02± 0.09] |
| Temporal cortex | 0.27± 0.06 |  | 0.28± 0.06 |  | 0.02± 0.05 |  | 15 |  | 1.33 |  | 0.204 |  | [-0.01± 0.04] |
| ACC | 0.44± 0.09 |  | 0.51± 0.16 |  | 0.07± 0.1 |  | 15 |  | 2.71 |  | 0.016 |  | [0.01± 0.12] |
| Amygdala | 0.96± 0.15 |  | 1.02± 0.14 |  | 0.06± 0.15 |  | 15 |  | 1.66 |  | 0.119 |  | [-0.02± 0.14] |
| Caudatus | 0.78± 0.19 |  | 0.85± 0.21 |  | 0.07± 0.15 |  | 15 |  | 1.92 |  | 0.074 |  | [-0.01± 0.15] |
| Hippocampus | 0.44± 0.1 |  | 0.5± 0.1 |  | 0.06± 0.08 |  | 15 |  | 3.01 |  | 0.009 |  | [0.02± 0.11] |
| Insula | 0.56± 0.09 |  | 0.61± 0.15 |  | 0.05± 0.09 |  | 15 |  | 2.3 |  | 0.036 |  | [0± 0.1] |
| Pallidum | 0.99± 0.19 |  | 1± 0.19 |  | 0± 0.18 |  | 15 |  | 0.1 |  | 0.921 |  | [-0.09± 0.1] |
| PCC | 0.41± 0.11 |  | 0.51± 0.17 |  | 0.1± 0.1 |  | 15 |  | 3.76 |  | 0.002 |  | [0.04± 0.15] |
| Putamen | 1.14± 0.14 |  | 1.22± 0.18 |  | 0.08± 0.14 |  | 15 |  | 2.13 |  | 0.05 |  | [0± 0.15] |
| Thalamus | 1.18± 0.19 |  | 1.28± 0.17 |  | 0.09± 0.16 |  | 15 |  | 2.42 |  | 0.029 |  | [0.01± 0.18] |
| Dorsal Raphe | 2.98± 0.59 |  | 3.16± 0.57 |  | 0.18± 0.5 |  | 15 |  | 1.44 |  | 0.17 |  | [-0.09± 0.45] |
|  |  |  |  |  |  |  |  |  |  |  |  |  |  |
| ACC, Anterior cingulate cortex; BP_ND_, Non displacable binding potential; CI, Confidence interval; DF, Degrees of freedom;  MDD, Major depressive disorder; PCC, Posterior cingulate cortex; SD, standard deviation | | | | | | | | | | | | | |

| **Supplementary Table 3.** [^11^C]MADAM data, patients with MDD and healthy controls (n=17 per cell) | | | | | | | | | | | | | |
| --- | --- | --- | --- | --- | --- | --- | --- | --- | --- | --- | --- | --- | --- |
| *Region* | *Patients (BP_ND_)* |  | *Controls (BP_ND_)* |  | *Difference* |  | *Paired t-test* | | | | | | |
|  |  |  |  |  |  |  |  |  |  |  |  |  |  |
|  | *Mean ± SD* |  | *Mean ± SD* |  | *Mean ± SD* |  | DF |  | t statistic |  | p value |  | CI (95%) |
|  |  |  |  |  |  |  |  |  |  |  |  |  |  |
| Frontal cortex | 0.23± 0.08 |  | 0.22± 0.04 |  | -0.01± 0.08 |  | 16 |  | -0.53 |  | 0.601 |  | [-0.05± 0.03] |
| Occipital cortex | 0.28± 0.08 |  | 0.27± 0.05 |  | -0.01± 0.1 |  | 16 |  | -0.55 |  | 0.593 |  | [-0.06± 0.04] |
| Parietal cortex | 0.25± 0.08 |  | 0.23± 0.04 |  | -0.01± 0.09 |  | 16 |  | -0.65 |  | 0.523 |  | [-0.06± 0.03] |
| Temporal cortex | 0.27± 0.06 |  | 0.26± 0.04 |  | 0± 0.06 |  | 16 |  | -0.34 |  | 0.74 |  | [-0.04± 0.03] |
| ACC | 0.45± 0.09 |  | 0.4± 0.06 |  | -0.05± 0.1 |  | 16 |  | -1.99 |  | 0.064 |  | [-0.09± 0] |
| Amygdala | 0.96± 0.14 |  | 0.93± 0.12 |  | -0.03± 0.15 |  | 16 |  | -0.72 |  | 0.482 |  | [-0.1± 0.05] |
| Caudatus | 0.79± 0.2 |  | 0.73± 0.12 |  | -0.06± 0.16 |  | 16 |  | -1.5 |  | 0.153 |  | [-0.14± 0.02] |
| Hippocampus | 0.44± 0.1 |  | 0.38± 0.08 |  | -0.06± 0.11 |  | 16 |  | -2.18 |  | 0.044 |  | [-0.11± 0] |
| Insula | 0.56± 0.09 |  | 0.54± 0.07 |  | -0.03± 0.1 |  | 16 |  | -1.15 |  | 0.267 |  | [-0.08± 0.02] |
| Pallidum | 1± 0.19 |  | 0.92± 0.16 |  | -0.07± 0.21 |  | 16 |  | -1.46 |  | 0.164 |  | [-0.18± 0.03] |
| PCC | 0.42± 0.11 |  | 0.39± 0.07 |  | -0.03± 0.12 |  | 16 |  | -0.91 |  | 0.376 |  | [-0.09± 0.04] |
| Putamen | 1.15± 0.14 |  | 1.12± 0.15 |  | -0.03± 0.17 |  | 16 |  | -0.83 |  | 0.419 |  | [-0.12± 0.05] |
| Thalamus | 1.19± 0.18 |  | 1.17± 0.1 |  | -0.01± 0.19 |  | 16 |  | -0.32 |  | 0.75 |  | [-0.11± 0.08] |
| Dorsal Raphe | 3.07± 0.57 |  | 3.15± 0.42 |  | 0.08± 0.67 |  | 16 |  | 0.46 |  | 0.648 |  | [-0.27± 0.42] |
|  |  |  |  |  |  |  |  |  |  |  |  |  |  |
| ACC, Anterior cingulate cortex; BP_ND_, Non displacable binding potential; CI, Confidence interval; DF, Degrees of freedom;  MDD, Major depressive disorder; PCC, Posterior cingulate cortex; SD, standard deviation | | | | | | | | | | | | | |


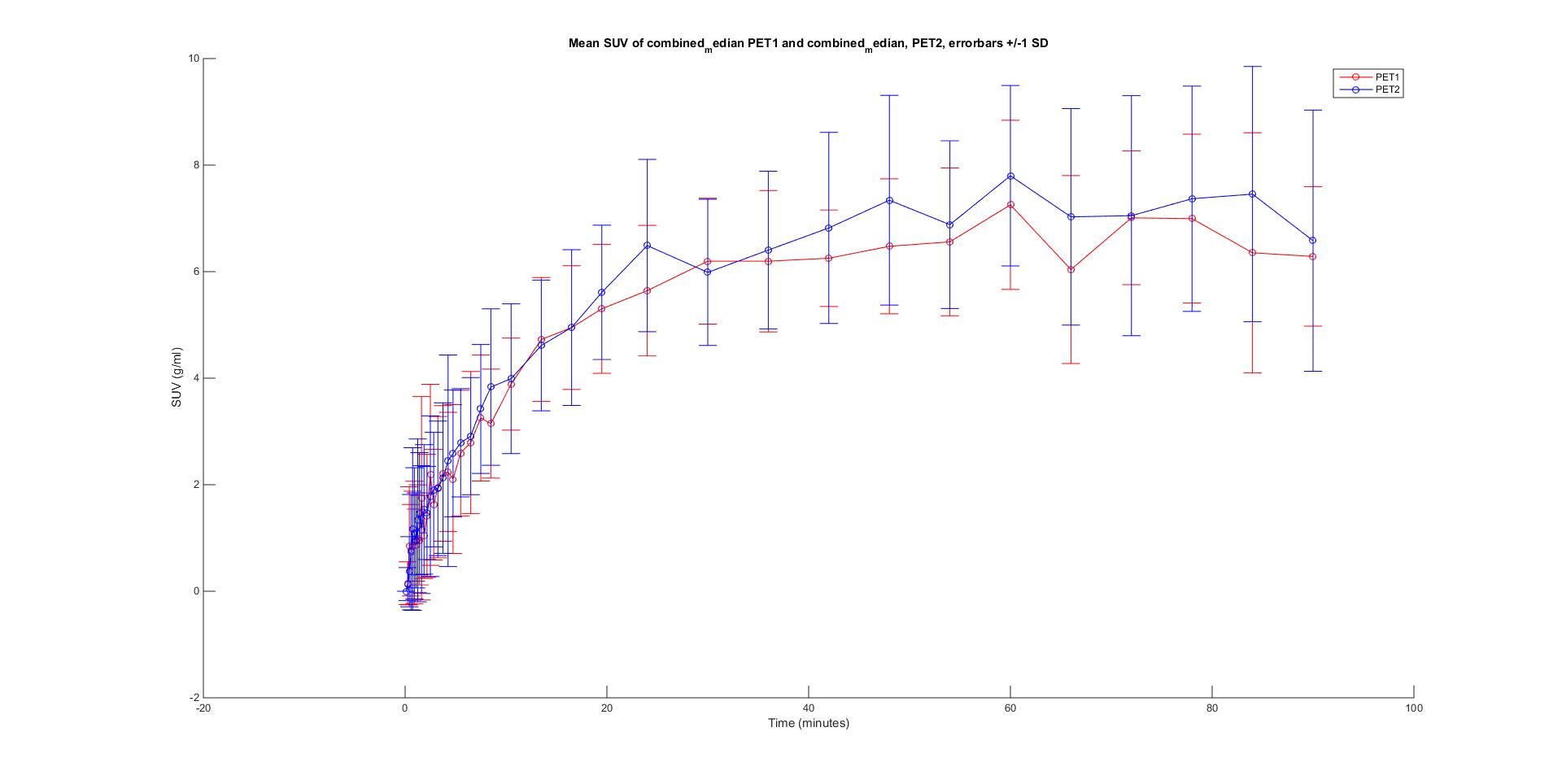


**Supplementary Figure 3**. Average standard uptake value time activity curve for median raphe for all patients, PET1 in red and PET2 in blue


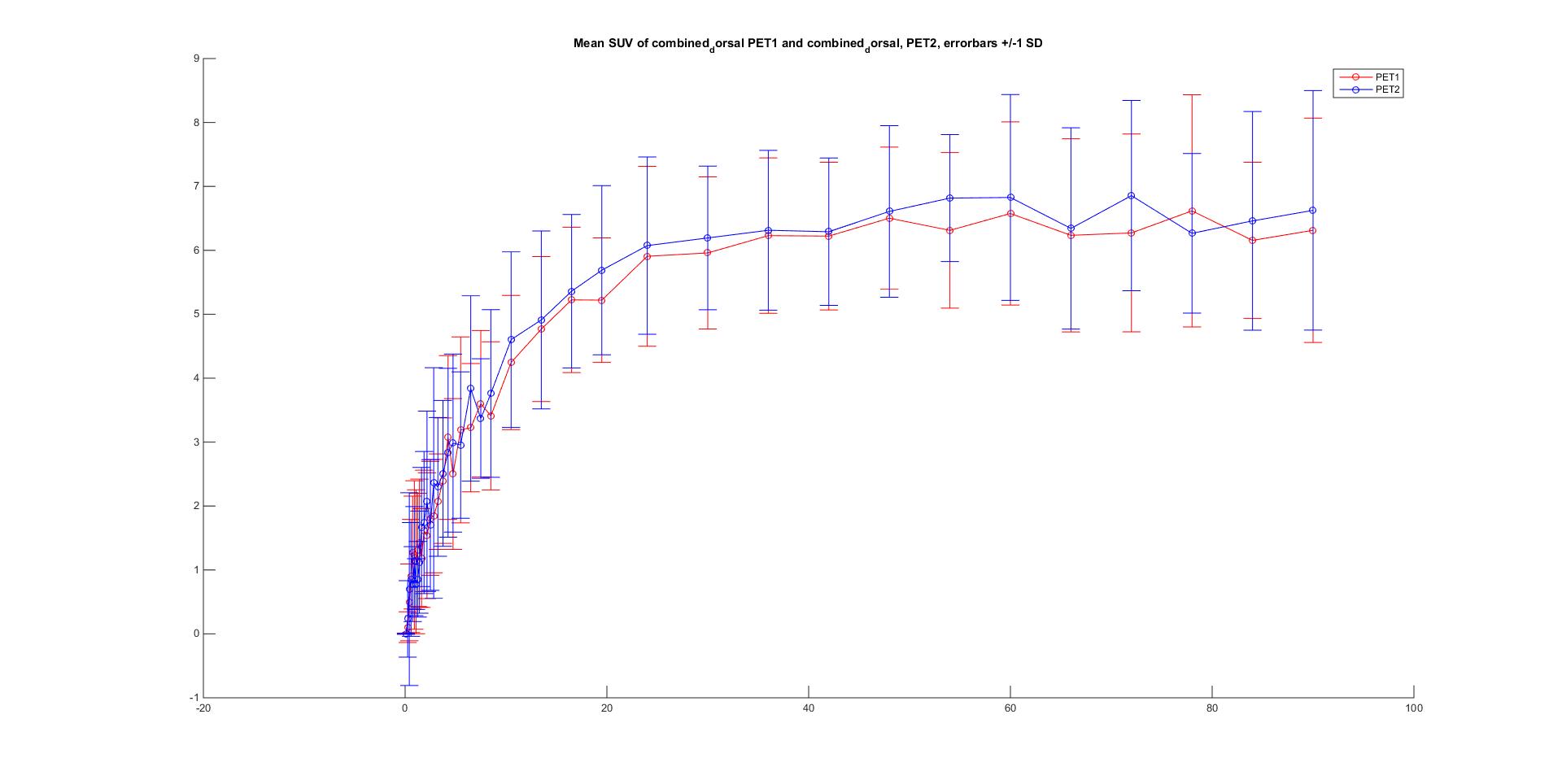


**Supplementary Figure 4**. Average standard uptake value time activity curve for dorsal raphe for all patients, PET1 in red and PET2 in blue

References

Beck, A.T., Ward, C.H., Mendelson, M., Mock, J., Erbaugh, J., 1961. An Inventory for Measuring Depression. Arch. Gen. Psychiatry 4, 561–571. https://doi.org/10.1001/archpsyc.1961.01710120031004

Beliveau, V., Svarer, C., Frokjaer, V.G., Knudsen, G.M., Greve, D.N., Fisher, P.M., 2015. Functional connectivity of the dorsal and median raphe nuclei at rest. Neuroimage 116, 187–195. https://doi.org/https://doi.org/10.1016/j.neuroimage.2015.04.065

Busner, J., Targum, S.D., 2007. The clinical global impressions scale: applying a research tool in clinical practice. Psychiatry (Edgmont). 4, 28–37.

Iglesias, J.E., Van Leemput, K., Bhatt, P., Casillas, C., Dutt, S., Schuff, N., Truran-Sacrey, D., Boxer, A., Fischl, B., 2015. Bayesian segmentation of brainstem structures in MRI. Neuroimage 113, 184–195. https://doi.org/https://doi.org/10.1016/j.neuroimage.2015.02.065

Montgomery, S.A., Åsberg, M., 1979. A New Depression Scale Designed to be Sensitive to Change. Br. J. Psychiatry 134, 382–389. https://doi.org/DOI: 10.1192/bjp.134.4.382

Svanborg, P., Åsberg, M., 1994. A new self-rating scale for depression and anxiety states based on the Comprehensive Psychopathological Rating Scale. Acta Psychiatr. Scand. 89, 21–28. https://doi.org/10.1111/j.1600-0447.1994.tb01480.x
