# Supplementary material for: Serotonin transporter availability increases in patients recovering from a depressive episode
Source: Transl Psychiatry. 2021 May 10;11:264. doi: 10.1038/s41398-021-01376-w (PMC8110529; doi:10.1038/s41398-021-01376-w)
Supplement: Supplementary file 2 — Supplement 2 [file 41398_2021_1376_MOESM2_ESM.pdf]

AsPredicted
Pre-Registration made easy

CONFIDENTIAL - FOR PEER-REVIEW ONLY

KIH14001_[11C]MADAM_PET (#14118)

Created: 09/17/2018 06:47 AM (PT)
Shared: 09/23/2018 11:38 PM (PT)

This pre-registration is not yet public. This anonymized copy (without author names) was created by the author(s) to use during peer-review. A non-anonymized version (containing author names) will become publicly available only if an author makes it public. Until that happens the contents of this pre-registration are confidential.

1) Have any data been collected for this study already?
It's complicated. We have already collected some data but explain in Question 8 why readers may consider this a valid pre-registration nevertheless.

2) What's the main question being asked or hypothesis being tested in this study?
A) The concentration of serotonin transporter in the brain will change in patients with depression after treatment with cognitive behavioral therapy (CBT).

B) Depressed patients will differ in concentration of serotonin transporter compared to matched healthy controls.

3) Describe the key dependent variable(s) specifying how they will be measured.
The radioligand [11C]MADAM binds specifically to the serotonin transporter and will be measured with positron emission tomography (PET). Quantification will be done using logan graphical analysis with cerebellar cortex as reference region. The reported parameter will be non displaceable binding potential (BPND).

4) How many and which conditions will participants be assigned to?
All patients will be treated with internet guided CBT for depression according to a standardized protocol. Patients are examined twice with PET, at baseline and after CBT treatment. Controls will not receive any treatment and will be examined once.

5) Specify exactly which analyses you will conduct to examine the main question/hypothesis.
Confirmatory analysis: We will test two regions of interest (ROIs): A) a composite region consisting of: amygdala, anterior cingulate gyrus, posterior cingulate gyrus, caudate, hippocampus, insula cortex, putamen, thalamus, globus pallidum. B) The median raphe nucleus.

The composite region will be created using standardization. A vector of BPND values will be created for each ROI containing all examinations for that region (i.e. 34, two from each subject in the longitudinal analysis). The vector will be standardized. For each examination (e.g., subject 1, PET1) there will then be nine standardized measurements, one for each ROI. A weight will be applied (see below) to each region after which the mean z-score will be calculated. This will result in one averaged z-score per examination.

Weights for the composite region will be created using a parametric image (WAPI). For each ROI the following parameters will be extracted: number of voxels (N); mean BPND; standard deviation of the BPND estimate (SD). The standard error of the mean (SE) will be calculated according to: SD/ √ N. The weight for each ROI will be: mean BPND /SE, this approach takes both SNR and volume in to account.

FreeSurfer will be used for delineation of all the ROIs described above, as well as for the reference region. Sub regions of the raphe nuclei will be created using a semi-automatic method. A MNI-template for raphe will be warped to each PET-examination (a time-weighted summated PET-image); within this region the voxel with the highest BPND will be identified and in an iterative process the voxel with highest BPND adjacent to the initial voxel will be added to the ROI. This process will continue until a preset number of voxels is reached. For the longitudinal analysis the an average ROI will be created in MR-space and used for both examinatinons. For the cross sectional analysis either the “raw” PET-space ROIs will be used or a new MNI template will be created.

Statistics: Paired t-test will be used for all confirmatory analysis. For the raphe regions no prediction of direction of change is made. For the composite ROI the prediction in the cross-sectional analysis is higher binding in controls compared to patients. For the longitudinal analysis no prediction of direction is made for the composite ROI. The cross-sectional test of the composite region will be one sided while all other tests will be two sided. Thus two confirmatory tests will be performed per dataset. We assume a priori that the change in the composite ROI and in median raphe will be correlated and we will therefore not correct for multiple comparisons. If however the data does not support this belief (non-significant correlation or significant correlation below 0.4) we will adjust the alpha for multiple comparisons.

6) Describe exactly how outliers will be defined and handled, and your precise rule(s) for excluding observations.
Due to the relative small sample size it will be hard to identify statistical outliers with certainty. However, if we see negative BPND-values in any of the individual regions that are part of the composite region the modeling will have failed and the region will be excluded from further testing in the subject and the matching control subject. If more than two subjects show negative BPND in the same region the region will be excluded in all subjects.
If we discover bad data that cannot be corrected (e.g. excessive head movement, faulty head position in the camera) that subject will be excluded.

The final decision regarding this will be made by an experienced researcher blinded and not involved in the primary image analysis.

7) How many observations will be collected or what will determine sample size? No need to justify decision, but be precise about exactly how the number will be determined.
17 subjects and 17 controls (matched for age, gender, IQ) have already been recruited. This number was decided beforehand based on power considerations.

8) Anything else you would like to pre-register? (e.g., secondary analyses, variables collected for exploratory purposes, unusual analyses planned?)
Data gathering is finished when this preregistration is created. However no analysis has been performed. For PET-data it impossible to have any indication of the results before analysis has started.

To get a point estimate of the average change in the composite ROI the median ratio PET1/PET2 for all ROIs (using the same weights as described above) will be calculated for each subject/matched pair and then averaged.

If one or both of the hypothesis testing analysis (A and B above) yield non-significant results we will use a likelihood ratio based approach in an attempt to quantify the evidence for H0. Four alternative hypothesis (H1,H2,H3,H4) corresponding to -15%,-7.5%,+7.5%, and +15% difference between groups (either pre-post or control-patient) will be tested against H0 (no difference between groups).

We plan to perform several exploratory analyses. All ROIs listed under pt 5) will be individually analyzed. Additionally we will look at the dorsal raphe and at cortical regions. Further we will explore the correlation between BPND in relevant ROIs to symptom ratings (QUIDS, MADRS). We will also examine the correlation of change in symptom rating to the change in [11C]MADAM binding. Further we will attempt to predict the response to CBT from baseline BPND. We have also gathered actigraphy data and will compare outcomes from this to the BPND data. The result from these analyzes will clearly be labeled as exploratory in the final paper. P values will be reported but no alpha will be set for error control.
